# Italian National Surveillance of Alcohol-Based Hand Rub Consumption in a Healthcare Setting—A Three-Year Analysis: 2020–2022

**DOI:** 10.3390/jcm13123371

**Published:** 2024-06-07

**Authors:** Alessandra Caramia, Daniele Petrone, Claudia Isonne, Francesco Battistelli, Sauro Sisi, Stefano Boros, Giulia Fadda, Maria Fenicia Vescio, Adriano Grossi, Martina Barchitta, Valentina Baccolini, Patrizio Pezzotti, Fortunato D’Ancona

**Affiliations:** 1 Department of Medical and Surgical Sciences, University of Foggia, 71121 Foggia, Italy; 2 Department of Infectious Diseases, Istituto Superiore di Sanità, 00162 Rome, Italy; 3 Department of Public Health and Infectious Diseases, Sapienza University of Rome, 00185 Rome, Italy; 4 Department of Life, Health and Environmental Sciences, University of L’Aquila, 67100 L’Aquila, Italy; 5 Department of Prevention, South-East Tuscany Local Health Authority, 52100 Arezzo, Italy; 6 Department of Medical and Surgical Sciences and Advanced Technologies “GF Ingrassia”, University of Catania, 95123 Catania, Italy

**Keywords:** hand hygiene, alcohol-based hand rub, healthcare-associated infections, national surveillance system

## Abstract

**Background/Objectives:** Hand hygiene (HH) is pivotal in mitigating infectious disease transmission and enhancing public health outcomes. This study focuses on detailing the national surveillance system for alcohol-based hand rub (ABHR) consumption in healthcare facilities across Italy, presenting results from a comprehensive three-year evaluation period, from 2020 to 2022. It aims to delineate this surveillance system and report on ABHR consumption trends in various Regions/Autonomous Provinces (Rs/APs). **Methods:** ABHR consumption data, collected through the ABHR Italian national surveillance system, coordinated by the Istituto Superiore di Sanità (ISS), were analyzed. Statistical methods, e.g., the Mann–Whitney test, were used to assess trends in ABHR consumption, expressed in liters per 1000 patient days (L/1000PD). **Results:** The results show significant variation in ABHR consumption across Rs/APs and over the years studied. National median ABHR consumption decreased from 2020 to 2022, with a significant reduction from a median of 24.5 L/1000PD in 2020 to 20.4 L/1000PD in 2021 and 15.6 L/1000PD in 2022. **Conclusions:** The decline in ABHR consumption raises concerns about the ongoing adherence to HH practices in Italian healthcare settings. This underscores the essential role that systematic ABHR monitoring and improved surveillance play in enhancing HH compliance, suggesting that sustained and strategic efforts are fundamental to uphold high standards of hygiene and to effectively respond to fluctuating ABHR usage trends over time. Further research is needed to explore barriers to effective ABHR use and to develop targeted strategies to improve HH practices.

## 1. Introduction

Hand hygiene (HH) is a key component of public health strategies aimed at reducing the transmission of infectious diseases and improving overall population health [1,2]. It has a crucial role in preventing healthcare-associated infection (HAI) onset, with the contaminated hands of healthcare workers being the main vehicle for microorganisms’ transmission [3,4]. For this reason, HH is considered one of the most effective infection prevention and control (IPC) measures in a healthcare context, preventing up to 50% of avoidable HAIs and helping counteract the spread of antimicrobial resistance (AMR) [5]. Furthermore, several economic evaluations show that the promotion of HH is a cost-effective intervention [6]. Its importance is evidenced by the World Health Organization’s (WHO) establishment of the “SAVE LIVES: Clean Your Hands” campaign that seeks to advance the objective of implementing hand hygiene in healthcare settings on a global scale [7].

Representing one the most effective ways of preventing the cross-transmission of microorganisms, the WHO has also developed an evidence-based guideline to support and facilitate the implementation and evaluation of a strategy to improve HH [8]. Among them, the use of alcohol-based hand rub (ABHR) is highly recommended for routine hand sanitization in healthcare areas, especially for its easy availability, the independence from a clean water source, and the proven effectiveness in reducing the microbial load [8]. To date, many healthcare facilities around the world already have well-established policies and guidelines aimed at HH improvement, including the use of ABHR at the point of care. In addition, studies showed that the COVID-19 pandemic has increased the use of ABHR, also improving healthcare workers’ compliance with HH [9]. However, HH remains a longstanding challenge due to concerns linked to the adherences of healthcare workers (HCWs) and the infrastructures and resources needed to focus on its enhancement [10].

Within this context, HH monitoring represents an important piece of the strategy. The direct observation based on the WHO protocol remains the gold standard to evaluate HCW compliance [11]; however, due to some difficulties in its implementation, the consumption of ABHR is used as a surrogate parameter to measure the adherence to HH guidelines in hospitals [12].

In Italy, the importance of establishing a monitoring system for the consumption of the hydroalcoholic solution for HH was underlined as a priority by the National Plan against Antibiotic Resistance (PNCAR) 2022–2025 and by the Italian National Plan of Prevention 2020–2025 [13,14].

In 2021, the first “National surveillance of the consumption of hydroalcoholic solution for HH in hospitals” in Italy was established with the aim to promote the strategies of implementation, promotion, and control of correct hand sanitation practices [15]. The aim of this study is to describe the Italian surveillance system for the hydroalcoholic HH solution in healthcare settings at the national level and to provide the results of three years of surveillance.

## 2. Materials and Methods

### 2.1. Surveillance System and Data Collection

In this study, we analyzed data retrieved from the National Surveillance of the consumption of hydroalcoholic solution for hand hygiene in hospitals. This system was established in 2021, and it is coordinated at the national level by the Epidemiology, Biostatistics and Mathematical Models unit of the Department of Infectious Diseases of the Istituto Superiore di Sanità (ISS, i.e., the Italian National Institute of Health) and involves the collection of data on ABHR consumption from Regions/Autonomous Provinces (Rs/APs) that are in charge of collecting data from all the public and private hospitals. The participation of the Rs/APs is voluntary, even though it is requested by the PNCAR and the National Prevention Plan. It is important to note that participation is not uniform across all Rs/APs. As a result, the data gathered may not ensure complete representativeness at the local, regional, or national level. The surveillance system includes the collection of data on a half-yearly and/or annual basis on ABHR consumption expressed in liters and patient days (PD), which is the number of days of ordinary hospitalization. In both cases, the source of information was also required. The Rs/APs were invited to provide this information when data were available, stratified by three different levels: for the entire facility (first level), for the inpatient ordinary (second level), and for specific hospitalization areas (third level) that were grouped in medical, surgical, intensive care, emergency, orthopedic-traumatological, and other areas (child neuropsychiatry, ophthalmology, otolaryngology, psychiatry, large pediatric burns, large burns, recovery and functional rehabilitation, long-term patients, neonatology, and rheumatology). The second level excludes any service or treatment that does not require hospitalization (such as day hospital and outpatient care) and includes only inpatient ordinary in the various wards. This strategy was aimed at collecting data from all the structures, defining a roadmap toward detailed data collection. Data retrieved from hospitals and health facilities were collected by the Regional/AP coordinators, previously identifying which of them participated in surveillance, and were sent to the Italian National Institute of Health using preset Microsoft Excel^®^ files. In addition, the “CSIA-ISS” web application “https://csia.iss.it/” (Accessed on 16 February 2024) was implemented in March 2023 for data collection for 2022. 

According to the WHO, the consumption of hydroalcoholic solution was expressed in liters of hydroalcoholic solution consumed per 1000 days of ordinary hospitalization (L/1000PD) [16]. Based on data availability, the consumption was collected for each level for the years 2020, 2021, and 2022. We treated consumption values of zero as missing. To exclude excessively high consumption data, potentially due to reporting errors by the structures, we applied a cut-off based on the data distribution. This cut-off value varied according to the hospital area details; it was set to 200 L/1000PD for inpatient ordinary areas, 300 L/1000PD for surgical and orthopedic areas, and 400 L/1000PD for the entire facility, including intensive care and emergency areas. Based on previous studies [17], we considered 20 L/1000PD to be the desirable ABHR consumption value for inpatient ordinary areas. 

### 2.2. Statistical Analysis 

For this study, data from second-level (inpatient ordinary) and third-level (medical, surgical, and intensive care) areas were considered. The analysis included all Microsoft Excel^®^ files sent to ISS by the Rs/APs before the web application was implemented, and all the data were uploaded to it until 27 November 2023. Only inpatient ordinary data were included due to their representativeness, while data from other third-level areas were excluded due to insufficient information and because the highest number of healthcare-associated infections (HAIs) were observed in the included areas. Semester data were treated as annual for hospitals that only submitted those.

We analyzed ABHR consumption at regional and national levels for inpatient ordinary care annually using plots and descriptive statistics (median value and interquartile range (IQR)). We also performed graphical and statistical evaluations (using the Mann–Whitney test) to assess the yearly median consumption trends for hospitals that provided data for all three years, aggregated by Region/AP. A sensitivity analysis was conducted on the same hospitals using a linear regression model with mixed effects, stratified by Region/AP, with the year as a fixed effect and the structure code as a random effect. Additionally, we performed a descriptive analysis for third-level areas at regional and national levels. In tables and plots, Rs/APs were ordered according to the numerical identification code provided by the Italian Institute of Statistics [18]. All analyses were conducted using RStudio 2023.03.0 under R 4.3.0. [19].

## 3. Results

In Italy, consumption data about inpatient ordinary (second level) were provided from 304 hospitals in 2020, 344 in 2021, and 549 hospitals in 2022 (Table 1).

**Figure 1:**
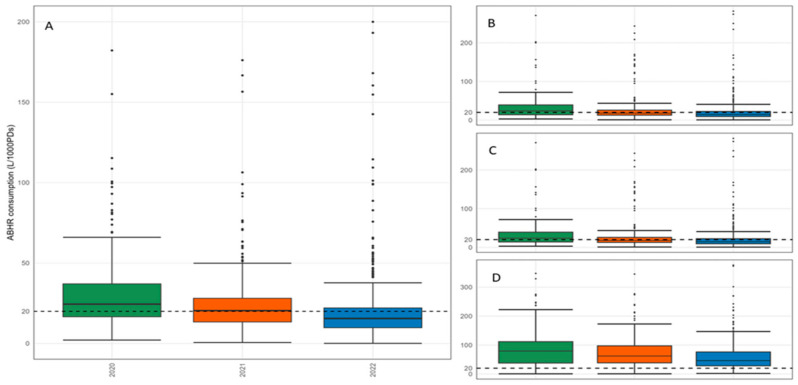
Italian ABHR consumption expressed in L/1000PDs: second level—inpatient ordinary (**A**), third level—medical (**B**), surgical (**C**), and intensive care (**D**) areas in 2020, 2021, and 2022.

In 2020, 2 structures sent data only for a semester, and the number increased in 2022, when only one semester’s data were sent by 42 hospitals. We set the missing values of consumption to zero for eight records (i.e., yearly data from a structure) for inpatient ordinary data. The national median ABHR consumption for inpatient ordinary area was 24.5 L/1000PD (IQR, 16.6–37.2 L/1000PD) in 2020, 20.4 L/1000PD (IQR, 13.4 L/1000PD–28.2 L/1000PD) in 2021, and 15.6 L/1000PD (IQR, 9.8 L/1000PD–22.1 L/1000PD) in 2022. (Table 1). As shown in Figure 1 and Table 1, in 2020 and 2021, the median ABHR consumption was above the threshold of 20 L/1000PD, while in 2022, it was just below the threshold. In addition, a wider IQR and, thus, greater variability in ABHR consumption in 2020 was observed, while in 2022, the reported ABHR consumption decreased, with less dispersion. The number of outlier values was about the same for the three years. 

**Figure 2:**
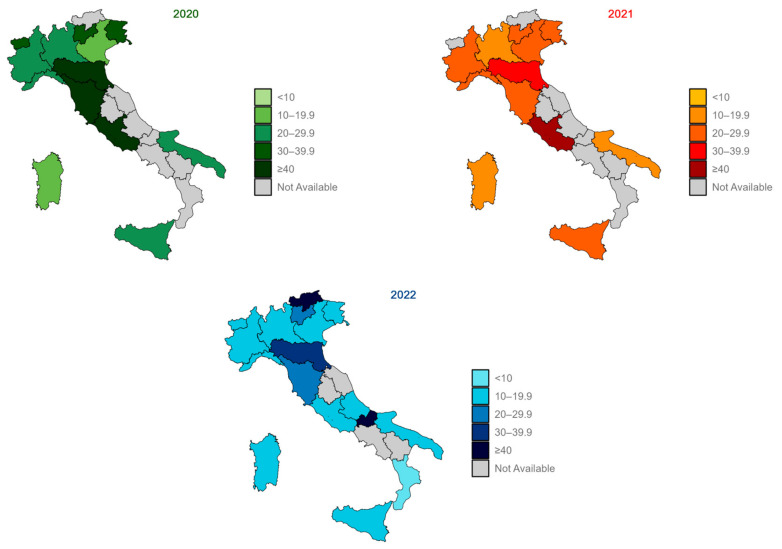
Median ABHR consumption in Rs/Aps: second-level—inpatient ordinary in 2020, 2021, and 2022.

ABHR consumption data submissions regarding the inpatient ordinary area were received from 13 Rs/APs in 2020 (Piedmont, Aosta Valley, Lombardy, A.P. of Trento, Veneto, Friuli-Venezia Giulia, Liguria, Emilia-Romagna, Tuscany, Lazio, Apulia, Sicily, and Sardinia), followed by 12 submissions in 2021 (Piedmont, Lombardy, A.P. of Trento, Veneto, Friuli-Venezia Giulia, Liguria, Emilia-Romagna, Tuscany, Lazio, Apulia, Sicily, and Sardinia). Remarkably, in 2022, there was an increase in the number of participating Rs/Aps, with a greater involvement of central Italian regions and data submitted by a total of 17 Rs/APs (Piedmont, Aosta Valley, Lombardy, A.P. of Bolzano, A.P. of Trento, Veneto, Friuli-Venezia Giulia, Liguria, Emilia-Romagna, Tuscany, Lazio, Abruzzo, Molise, Apulia, Calabria, Sicily, and Sardinia). In Italy, as shown in Figure 2 and Table 1, the median ABHR consumption varied between Rs/APs, also changing in the different years, i.e., 2020, 2021, and 2022. In 2020, three Rs/APs were represented in the highest interval (≥40 L/1000PD). This number decreases in 2021, where only one region was above 40 L/1000PD. Finally, in 2022, the number of Rs/APs increased to two. In both 2020 and 2021, the majority of the Rs/APs that participated in the surveillance reported an ABHR consumption higher than the threshold (20 L/1000PD); by contrast, in 2022, less than 33 per cent of the participating regions (5/17) were represented in the same level.

Out of the total number of participating Rs/APs, only 12 of them had at least one hospital that provided inpatient ordinary data for each year considered in the analysis, for a total of 236 hospitals. In Italy, significant decreases in ABHR consumption in the inpatient ordinary (*p* ≤ 0.05) area were recorded during the study period (2020 to 2021, 2021 to 2022) and in the 2020–2022 comparison (Table 2).

**Figure 3:**
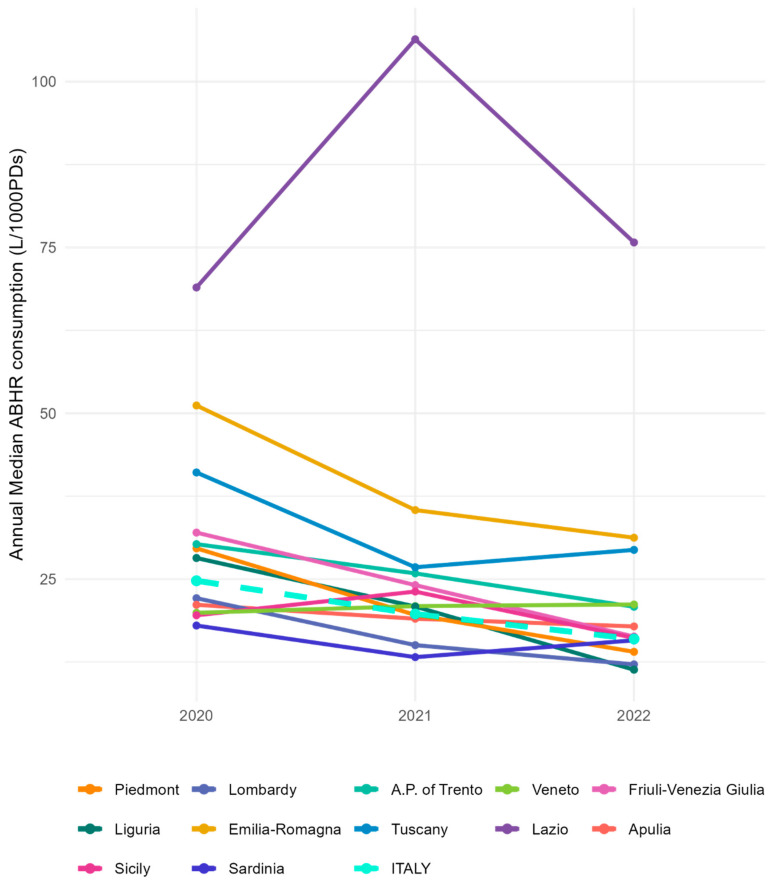
Graphical trend analysis of regional and national aggregated data for hospitals that sent ABHR consumption data for all three years (2020, 2021, and 2022)—inpatient ordinary, Italy.

At the regional level, seven Rs/APs had a decrease in consumption in both periods (2020 to 2021 and 2021 to 2022), and only one region had an increase in consumption. A notable reduction in ABHR consumption (*p* ≤ 0.05) was observed across seven Rs/APs: Piedmont, Lombardy, A.P. of Trento, Friuli-Venezia Giulia, Liguria, Emilia-Romagna, and Apulia during the comparison between 2020 and 2021. Similarly, in the comparison between 2021 and 2022, a significant reduction in ABHR consumption was observed across five Rs/APs (*p* ≤ 0.05): Piedmont, Lombardy, Friuli-Venezia Giulia, Liguria, and Emilia-Romagna. Furthermore, when comparing 2020 and 2022, significant reductions in ABHR consumption (*p* ≤ 0.5) were evident in seven Rs/APs: Piedmont, Lombardy, A.P. of Trento, Friuli-Venezia Giulia, Liguria, Emilia-Romagna, and Apulia. Notably, only the Veneto region reported an increase in ABHR consumption during both the 2020–2021 and 2020–2022 comparisons, but the increase was not significant, as the rare increases in consumption observed in other Rs/Aps (Figure 3). Except for the region of Lazio, for which the model could not be estimated, the sensitivity analysis based on the statistical model showed no major differences from the main analysis based on the Mann–Whitney test (Table S1).

If we had zero values, we set them to missing for four records (i.e., yearly data of a hospital) for the medical area data, six records for the surgical area data, and six records for the IC area data. In Italy, as shown in Figure 2 and Table 1, the median ABHR consumption in both the medical and surgical areas was over 20 L/1000PD in 2020 (20.3 and 23.8 L/1000PD, respectively), and it was below the desirable value in 2021 and 2022; on the other hand, the median consumption in IC area was higher than the threshold in all the years included in the analysis (79.5 in 2020, 62.2 in 2021, and 46.1 L/1000PD in 2022). Not all the Rs/APs that communicated data for inpatient ordinary reported data for medical, surgical, and IC areas. Specifically, between 10 and 12 Rs/APs released data for the areas included in the analysis in 2020 and 2021, while 14 reported data for these areas in 2022. There was heterogeneity in the median ABHR consumption in medical and surgical areas between the different years. Notably, in medical areas in 2022, all the Rs/APs involved (n = 14) reported a median consumption of less than 20 L/1000PD. As observed at the national level, the consumption in IC areas was above the desirable value for all the years and all the Rs/APs that reported values for this area (Table S2). 

## 4. Discussion

The results of the national surveillance showed a reduction in the overall consumption of ABHR in healthcare settings from 2020 to 2022, reaching values even below the recommended ABHR consumption in 2022 (at least 20 L/1000PD [16]). With the onset of the pandemic and the need to implement strategies to counter the spread of SARS-CoV-2, HH regained a fundamental role and became one of the necessary actions to be taken in a healthcare context [20], so much so that all healthcare facilities had to be equipped with hydroalcoholic solution dispensers [21]. The abundant consumption recorded in 2020 may thus be a consequence of its extensive use in healthcare settings, which, following the recommendations of national and supranational institutions [22], promoted its use as a pillar strategy of prevention. On the other hand, the initial awareness-raising campaign toward health workers that took place at the start of the pandemic regarding the implementation of hand hygiene adherence also contributed positively to maintaining high consumption values [23]. As the pandemic continued, the shortage of ABHR [9] and less attention to prevention strategies, including HH by health workers, due to fatigue [24] and because the arrival of vaccines probably decreased the fear of infection [25], may explain the decrease in its consumption recorded in the surveillance system in 2021 and 2022. This highlights, on the one hand, the importance of maintaining high awareness of hand hygiene in health workers through training and awareness raising [26], and on the other hand, the critical role of governance in investing in preparedness strategies [27]. 

The decrease in the consumption of hydroalcoholic solution was recorded in all areas considered but with differences in the intensive care setting. Here, in contrast to the medical and surgical areas, although there was a decrease in its use, the consumption values of hydroalcoholic solution fortunately remained above the threshold value. The high values were in line with those provided in the latest report of the Italian Nosocomial Infections Surveillance in Intensive Care Units (SPIN-UTI) Project [28], with a median consumption of 106.7 L/1000PD in 2020. Effectively, during the pandemic, these wards, more than the others, had to face a large number of COVID-19 patients requiring care, and, therefore, perhaps to a greater extent than the others, had to apply measures to counter the spread of SARS-CoV-2 [29]. For this reason, a great deal of effort went into training healthcare personnel as well as trying to reallocate resources to provide the necessary tools to implement these measures [12]. The availability, among others, of hydroalcoholic solutions, together with awareness and training campaigns, may have fostered its use among healthcare workers in this setting [30]. However, even before the start of the pandemic, data on the consumption of hydroalcoholic solutions in Italy were not very encouraging, with some differences depending on the healthcare setting considered. According to the last European Centre for Disease Prevention and Control (ECDC) point prevalence survey (PPS) of healthcare-associated infections and antimicrobial use in acute care hospitals (PPS-HAIs) conducted in 2016–2017 [31], Italy was among the countries with the lowest ABHR consumption, with a median of 9.17 L/1000PD [32]. If we take into account that Italy is also one of the countries with a high prevalence of HAIs [33], there is an urgent need to focus efforts on the implementation of prevention and control measures in healthcare settings, including the promotion of hand hygiene.

Among the Italian regions, an increase in HH data reporting in surveillance systems was observed from 2020 to 2022. This result could be explained by the implementation of the data collection platform in 2022, which provided the regions with a greater ease of communicating data. Despite this, in 2022, there were still four regions (Umbria, Marche, Campania, and Basilicata) that had never recorded data on the consumption of hydroalcoholic solutions. Although complex, strengthening the use of surveillance platforms is a key factor in fostering participation in surveillance programs. At the same time, it is essential to try to increase awareness of the importance of collecting, communicating, and sharing data on the consumption of hydroalcoholic solutions [30] among healthcare professionals in order to monitor its progress over time and take any corrective actions. Despite the increase in the participation of the regions in surveillance, there was a general decreasing trend in the consumption of hydroalcoholic solutions from 2020 to 2022 among those who participated in all three years of data collection. In fact, although there were differences in the number of facilities involved between the various regions, there was a significant reduction in the consumption of hydroalcoholic solutions at the national level. Moreover, comparing 2020 to the other two years, the results of our study showed a significant reduction in 7 of the 12 participating regions, and the comparison between 2021 and 2022 also revealed similar results. As mentioned above, the factors associated with the pandemic that led to a strict application of prevention and control measures during the first phase, as opposed to a decline in attention and the availability of resources in the subsequent phases, may explain these findings [24]. However, it is noteworthy that in some regions, despite the significant reduction in consumption over the years, the median consumption values always remained above the threshold. These regions, starting from a high consumption in 2020, managed to maintain adequate consumption standards in the other two years as well. On the contrary, the only region in which there was an increase in consumption over the years maintained relatively low consumption values, reaching the value recommended by the WHO in only one year.

Lastly, some considerations need to be addressed regarding the use of hydroalcoholic solution consumption as a method of assessing adherence to hand hygiene. In Italy, as reported in the PNCAR, ABHR consumption surveillance represents the tool for indirectly monitoring HH, providing important data that can give useful feedback to the hospitals, highlight areas for improvement, and measure the effectiveness of targeted actions [15]. Although it is not the gold standard, the indirect method of calculating consumption was used because it allows quick and reliable mapping of the consumption of hydroalcoholic solutions at the national level [34]. In fact, the direct observation of HH practices, which is considered a gold standard according to the 2009 WHO guidelines, is a resource-intensive method requiring the continuous presence of observers at all stages of patient care [8]. This practice, especially for the challenges posed by the COVID-19 pandemic, in terms of staff reallocation and shortage of healthcare workers, would have made the monitoring of hand hygiene practice very difficult [35]. On the other hand, by focusing on the consumption of hydroalcoholic solutions, it was possible to quickly collect data using fewer resources and still determine the current consumption as well as also the trend over time.

This paper has several strengths and limitations. First, data on the consumption of hydroalcoholic solutions may not perfectly reflect the actual adherence to proper hand hygiene. Nevertheless, this method allowed for rapid and inexpensive data collection, which is particularly appropriate considering the pandemic period in which the collection took place. Second, since the surveillance was established in recent years, there may have been obstacles in the collection and transmission of data due to the initial inexperience of the operators. In spite of this, the subsequent use of the online platform has made it possible to have a facilitated and standardized collection. Another possible limitation of the survey is the voluntary and uneven participation in data collection across Rs/APs and facilities. As a result, the data may not fully represent all local, regional, or national levels, potentially introducing biases that could affect the generalizability of our results. Therefore, while the results provide valuable insights, they should be interpreted with caution. A further limitation is that it collected the volume of ABHR consumption bought but not strictly consumed by hospitals. It should also be emphasized that the evaluation of the use of ABHR has drawbacks, including its potential misuse for purposes such as surface disinfection, unnecessary HH actions, or use by non-health workers, patients, or relatives. Consequently, data on ABHR must be analyzed with caution. 

However, to the best of our knowledge, this is the first time in which the results of the national surveillance on the consumption of hydroalcoholic solutions are described, monitoring the trend over time and covering three consecutive years. In addition, having included the years of the pandemic, these data provide an overview of the application of proper hand hygiene in healthcare facilities during that period. Still, it is necessary to implement such surveillance, raising awareness in the regions and making data collection as easy as possible.

## 5. Conclusions

In conclusion, these data support the importance of ABHR monitoring through the national surveillance system, and improving surveillance will lead to better results. In addition, the surveillance system makes it possible to contextualize the actions taken and identify those to be implemented. The decrease in the consumption of ABHR in 2021 and, especially, in 2022 compared to 2020 reinforces the need to continue efforts to maintain and improve the high HH standards achieved during the pandemic, as well as to take measures to address any declines in the future. This includes the promotion of an HH culture, education on proper handwashing techniques, access to hydroalcoholic solutions, and the creation of healthcare environments that encourage adherence to these practices. 

## Figures and Tables

**Table 1:** Number of structures involved (*n*), median, interquartile ranges (IQR) of ABHR consumption expressed in L/1000PDs at national and regional level, inpatient ordinary. Italy, years 2020–2021–2022.

Region/AAPP	Area: 2nd Level			
	Year	2020	2021	2022
Piedmont	n	45	44	47
	Median (IQR)	29.8 (22.7–36.6)	20.6 (16.0–29.2)	14.5 (11.8–18.8)
Aosta Valley	n	1	-	1
	Median (IQR)	36.9 (36.9–36.9)	-	15.3 (15.3–15.3)
Lombardy	n	93	87	137
	Median (IQR)	21.6 (15.6–30.7)	14.8 (11.1–21.6)	12.1 (9.0–16.7)
A.P. of Bolzano	n	-	-	3
	Median (IQR)	-	-	44.5 (41.3–56.1)
A.P. of Trento	n	7	7	7
	Median (IQR)	30.3 (29.2–40.5)	25.9 (21.1–26.8)	20.8 (19.8–22.8)
Veneto	n	35	44	41
	Median (IQR)	18.3 (11.7–24.8)	20.2 (14.7–27.8)	19.1 (15.8–22.7)
Friuli-Venezia Giulia	n	12	13	16
	Median (IQR)	32.0 (24.6–38.8)	24.4 (22.7–27.4)	16.3 (13.1–22.3)
Liguria	n	15	14	15
	Median (IQR)	28.2 (22.7–39.5)	21.2 (13.9–22.3)	11.3 (9.2–18.5)
Emilia-Romagna	n	13	13	13
	Median (IQR)	55.5 (45.2–82.9)	33.8 (25.7–38.9)	31.2 (31.0–33.1)
Tuscany	n	17	45	44
	Median (IQR)	41.1 (20.7–59.2)	23.6 (13.4–32.8)	22.8 (17.6–25.7)
Lazio	n	4	1	81
	Median (IQR)	48.7 (33.2–61.2)	106.4 (106.4–106.4)	12.0 (4.9–21.8)
Abruzzo	n	-	-	16
	Median (IQR)	-	-	18.4 (15.5–23.1)
Molise	n	-	-	4
	Median (IQR)	-	-	42.1 (38.5–45.9)
Apulia	n	17	16	30
	Median (IQR)	24.3 (15.3–50.3)	19.0 (11.4–26.9)	18.1 (12.1–22.9)
Calabria	n	-	-	14
	Median (IQR)	-	-	9.8 (6.5–33.6)
Sicily	n	33	35	56
	Median (IQR)	20.8 (13.7–39.5)	23.2 (18.9–31.5)	13.2 (8.6–22.7)
Sardinia	n	12	25	24
	Median (IQR)	17 (10.6–22.5)	16.7 (12.6–21.9)	14.6 (8.6–19.5)
Italy	n	304	344	549
	Median (IQR)	24.5 (16.6–37.2)	20.4 (13.4–28.2)	15.6 (9.8–22.1)

Note: Four regions (Umbria, Marche, Campania, and Basilicata) never sent data and were thus not included in the analysis.

**Table 2:** Comparison of regional and national median consumption trends between the years and statistical significance of the Mann–Whitney test of ABHR consumption for structures that sent ABHR consumption data for all three years (2020, 2021, and 2022)—inpatient ordinary.

Region/AAPP	2020 vs. 2021	2021 vs. 2022	2020 vs. 2022	Number of Structures Involved
Piedmont	29.6 vs. 19.6 dec **	19.6 vs. 14.0 dec ***	29.6 vs. 14.0 dec ***	42
Lombardy	22.1 vs. 15.0 dec ***	15.0 vs. 12.1 dec ***	22.1 vs. 12.1 dec ***	72
A.P. of Trento	30.3 vs. 25.9 dec *	25.9 vs. 20.8 dec §	30.3 vs. 20.8 dec *	7
Veneto	19.9 vs. 20.9 inc §	20.9 vs. 21.2 inc §	19.9 vs. 21.2 inc §	25
Friuli-Venezia Giulia	32.0 vs. 24.1 dec *	24.1 vs. 16.3 dec **	32.0 vs. 16.3 dec ***	12
Liguria	28.2 vs. 20.9 dec **	20.9 vs. 11.3 dec *	28.2 vs. 11.3 dec ***	13
Emilia-Romagna	51.2 vs. 35.4 dec **	35.4 vs. 31.2 dec .	51.2 vs. 31.2 dec **	8
Tuscany	41.1 vs. 26.8 dec §	26.8 vs. 29.4 inc §	41.1 vs. 29.4 dec §	15
Lazio	69.0 vs. 106.4 inc §	106.4 vs. 75.8 dec §	69.0 vs. 75.8 inc §	1
Apulia	21.1 vs. 19.0 dec .	19.0 vs. 17.9 dec §	21.1 vs. 17.9 dec *	16
Sicily	19.5 vs. 23.1 inc §	23.1 vs. 16.1 dec §	19.5 vs. 16.1 dec §	15
Sardinia	18.0 vs. 13.2 dec §	13.2 vs. 15.8 inc §	18.0 vs. 15.8 dec §	10
Italy	24.7 vs. 19.7 dec ***	19.7 vs. 16.0 dec ***	24.7 vs. 16.0 dec ***	236

inc: increasing and dec: decreasing. ‘***’ means significant at 0.1%; ‘**’ means significant at 1%; ‘*’ means significant at 5%; ‘.’ means significant at exactly 5%; and ‘§’ means not significant. The red color represents a reduction in ABHR consumption, and the green color represents an increase in ABHR consumption.

## Data Availability

The data presented in this study are available on request from the corresponding author due to legal reasons.

## Supplementary Materials

The following supporting information can be downloaded at: https://www.mdpi.com/article/10.3390/jcm13123371/s1, Table S1: Estimated values and 95% confidence interval obtained from the sensitivity analysis for ABHR consumption for structures that sent ABHR consumption data for all three years (2020, 2021, and 2022)—inpatient ordinary; Table S2: Number of structures involved (n), median, and quartiles (p25–p75) of ABHR consumption expressed in L/1000PDs at national and regional levels in medical, surgical, and intensive care areas in Italy in 2020, 2021, and 2022.
