# Supplementary material for: Italian National Surveillance of Alcohol-Based Hand Rub Consumption in a Healthcare Setting—A Three-Year Analysis: 2020–2022
Source: J Clin Med. 2024 Jun 7;13(12):3371. doi: 10.3390/jcm13123371 (PMC11204833; doi:10.3390/jcm13123371)
Supplement: Supplementary file 1 [file jcm-13-03371-s001.zip › jcm-3015532-supplementary.pdf]

Journal of
Clinical Medicine

Table S1. Estimated values and 95% confidence interval obtained from the sensitivity analysis for ABHR consumption for structures that sent ABHR consumption data for all three years (2020, 2021, and 2022)—inpatient ordinary.

**Region/AAPP**	**2020 vs. 2021**	**2021 vs. 2022**	**2020 vs. 2022**	**Number of Hospitals Involved**
Piedmont	−8.3 (−12.7 to −3.9)	−7.2 (−9.3 to −5.0)	−7.7 (−9.6 to −5.9)	42
Lombardy	−2.8 (−8.9 to 3.3)	−8.3 (−14.2 to −2.4)	−5.6 (−8.1 to −3.0)	72
A.P. of Trento	−6.1 (−10.3 to −2.0)	−4.3 (−8.3 to −0.3)	−5.2 (−7.2 to −3.2)	7
Veneto	4.0 (−2.8 to 10.8)	−3.7 (−9.0 to 1.6)	0.2 (−2.8 to 3.2)	25
Friuli-Venezia Giulia	−9.1 (−14.5 to −3.8)	−6.6 (−9.2 to −4.0)	−7.9 (−10.2 to −5.5)	12
Liguria	−11.1 (−16.0 to −6.2)	−5.8 (−9.6 to −2.0)	−8.4 (−11.2 to −5.7)	13
Emilia-Romagna	−21.4 (−41.1 to −1.8)	−4.7 (−8.2 to −1.3)	−13.1 (−20.6 to −5.5)	8
Tuscany	−7.7 (−19.4 to 4.0)	−5.6 (−20.6 to 9.5)	−6.6 (−13.1 to −0.1)	15
Lazio	-	-	-	1
Apulia	−10.1 (−22.4 to 2.2)	−7.3 (−14.2 to −0.4)	−8.7 (−13.7 to −3.6)	16
Sicily	−0.4 (−5.0 to 4.1)	−6.6 (−23.8 to 10.6)	−3.5 (−10.8 to 3.7)	15
Sardinia	1.5 (−14.1 to 17.0)	−6.4 (−23.5 to 10.7)	−2.5 (−8.7 vs 3.7)	10
**Italy**	−4.9 (−7.5 to −2.3)	−6.8 (−9.3 to −4.4)	−5.8 (−7.1 to −4.6)	236

Note: For the Lazio region, there were not enough data for the model estimations.

Table S2. Number of structures involved (n), median, and quartiles (p25–p75) of ABHR consumption expressed in L/1000 PDs at national and regional levels in medical, surgical, and intensive care areas in Italy in 2020, 2021, and 2022.

Region/AAPP	Area	Medical Area			Surgical Area			IC Area		
	**Year**	**2020**	**2021**	**2022**	**2020**	**2021**	**2022**	**2020**	**2021**	**2022**
**Piedmont**	n	10	37	43	10	33	37	9	32	36
	Median (IQR)	27.9 (23.4–36.4)	20.6 (13.2–29.3)	13.8 (10.9–17.7)	21.9 (14.3–26.0)	19.7 (15.1–24.1)	14.6 (11.0–19.3)	92.9 (57.7–121.6)	65.8 (43.8–84.2)	41.8 (34.8–69.2)
**Aosta Valley**	n	1		1	1		1	1		1
	Median (IQR)	27.3 (27.3–27.3)		11.9 (11.9–11.9)	36.4 (36.4–36.4)		13.1 (13.1–13.1)	210.0 (210.0–210.0)		67.4 (67.4–67.4)
**Lombardy**	n	49	57	97	42	49	80	27	29	54
	Median (IQR)	17.0 (11.1–28.5)	12.5 (8.5–18.3)	10.9 (7.9–15.5)	22.9 (13.9–32.9)	16.2 (9.7–24.0)	12.1 (8.1–17.1)	78.2 (37.8–97.8)	52.0 (38.5–98.7)	53.2 (35.5–99. 7)
**A.P. of Trento**	n	7	7	7	7	7	7	2	2	2
	Median (IQR)	25.8 (19.3–33.8)	20.9 (19.2–23.2)	18.6 (18.0–22.4)	42.3 (26.9–66.1)	40.6 (21.2–41.7)	26.4 (17.6–37.7)	110.6 (98.6–122.5)	73.3 (40.2–106.3)	78.9 (74.9–83.0)
**Veneto**	n	29	38	36	28	34	33	25	32	30
	Median (IQR)	19.1 (11.3–24.5)	19.0 (12.2–29.2)	16.9 (12.2–23.0)	18.8 (11.0–28.0)	18.3 (14.4–33.9)	17.1 (14.2–23.5)	69.3 (37.4–94.6)	67.9 (45.4–125.2)	62.5 (30.7–76.0)
**Friuli-Venezia Giulia**	n	7	8	11	7	8	11	4	6	8
	Median (IQR)	21.2 (17.4–29.9)	21.6 (19.2–26.1)	14.8 (11.8–19.9)	25.2 (18.1–39.4)	17.7 (13.5–21.2)	17.9 (12.6–22.3)	86.9 (67.1–89.8)	68.4 (38.3–81.2)	45.0 (24.9–62.7)
**Liguria**	n	12	11	14	12	11	13	11	11	13
	Median (IQR)	20.6 (16.4–28.4)	16.3 (9.3–20.8)	9.6 (8.1–15.0)	26.0 (20.0–42.5)	17.7 (13.2–22.2)	12.6 (10.3–19.9)	105.1 (71.0–136.4)	62.8 (37.3–96.6)	37.7 (30.5–52.4)
**Tuscany**	n	16	31	40	14	27	34	8	18	25
	Median (IQR)	30.0 (17.7–49.4)	18.3 (10.9–33.0)	17.1 (12.5–22.8)	37.9 (15.0–48.3)	21.3 (13.4–41.8)	20.8 (13.4–29.5)	144.8 (78.3–299.7)	85.7 (65.8–125.9)	56.1 (39.9–95.3)
**Lazio**	n	2		40	3	1	37	2		24
	Median (IQR)	48.8 (42.7–54.9)		8.9 (6.6–17.4)	47.7 (41.2–69.0)	106.4 (106.4–106.4)	13. 9 (8.5–24.3)	107.1 (84.2–130.0)		37.1 (23.5–62.3)
**Abruzzo**	n			16			14			12

	Median (IQR)			12.0 (7.5–22.8)			19.0 (11.5–36.1)			80.1 (34.1–151.7)
**Apulia**	n	16	15	26	16	14	25	10	10	19
	Median (IQR)	15.3 (12.0–42.7)	16.1 (8.7–38.7)	13.5 (8.6–21.0)	18.7 (12.3–40.4)	12.4 (8.5–19.4)	12.4 (6.3–17.8)	48.0 (33.9–177.5)	43.1 (30.5–138.3)	44.7 (22.8–105.5)
**Calabria**	n			13			8			6
	Median (IQR)			8.8 (6.5–12.3)			12.8 (4.2–68.3)			25.6 (19.8–53.2)
**Sicily**	n	18	19	46	17	18	44	16	16	24
	Median (IQR)	19.6 (10.4–33.3)	20.4 (14.4–26.8)	12.6 (8.4–21.1)	14.2 (9.3–29.8)	15.5 (11.5–23.2)	14.5 (7.1–27.9)	49.6 (20.9–113.0)	59.5 (36.5–89.5)	41.1 (21.9–73.6)
**Sardinia**	n	11	21	24	10	16	16	7	12	13
	Median (IQR)	17.2 (10.8–29.0)	16.6 (11.0–20.5)	15.6 (7.3–20.4)	31.8 (20.0–71.4)	15.9 (12.1–25.1)	12.1 (8.3–15.6)	33.7 (21.5–111.1)	27.0 (17.7–60.7)	25.9 (16.7–32.2)
**Italy**	n	178	244	414	167	218	360	122	168	267
	Median (IQR)	20.3 (13.4–32.0)	18.1 (11.1–24.8)	13.2 (8.7–19.0)	23.8 (13. 9–39.3)	18.0 (12.8–26.0)	14.6 (9.3–22.5)	79.5 (37.8–112.0)	62.2 (38.5–97.8)	46.1 (28.1–76.3)

Note: Four regions (Umbria, Marche, Campania, and Basilicata) never sent data and were thus not included in the analysis.
